# Solubility Enhancement of Steviol Glycosides and Characterization of Their Inclusion Complexes with Gamma-Cyclodextrin

**DOI:** 10.3390/ijms12117529

**Published:** 2011-11-03

**Authors:** Mani Upreti, Ken Strassburger, You L. Chen, Shaoxiong Wu, Indra Prakash

**Affiliations:** 1 The Coca-Cola Company, P.O. Box 1734, Atlanta, GA 30301, USA; E-Mails: kstrassburger@coca-cola.com (K.S.); yochen@coca-cola.com (Y.L.C.); iprakash@coca-cola.com (I.P.); 2 Nuclear Magnetic Resonance Center, Emory University, 1515 Pierce Drive Atlanta, GA 30322, USA; E-Mail: swu@emory.edu

**Keywords:** steviol glycoside, cyclodextrin, solubility, DSC, FT-IR, Raman, NMR

## Abstract

Steviol glycosidesrebaudioside (reb) A, C and D have low aqueous solubilities. To improve their aqueous solubilities, inclusion complex of steviol glycosides, reb A, C and D and gamma cyclodextrin were prepared by freeze drying method and further characterized by means of differential scanning calorimetry, Fourier transform infrared spectroscopy and Raman spectroscopy. The effect of gamma cyclodextrin on chemical shifts of the steviol glycosides was also studied in proton NMR experiments as well as in solid state ^13^C CP/MAS NMR experiments. These results indicated that the steviol glycosides were clearly in inclusion complex formation with the gamma cyclodextrin which also results in solubility enhancement of these steviol glycosides. Phase solubility studies showed that amounts of soluble reb A, C and D increased with increasing amounts of gamma cyclodextrin indicating formation of 1:1 stoichiometric and higher order inclusion complexes.

## 1. Introduction

Steviol glycosides are isolated and extracted from the *Stevia rebaudiana* (Bertoni) plant (“stevia”) commercially cultivated in Japan, Singapore, Taiwan, Malaysia, South Korea, China, Israel, India, Brazil, Australia, and Paraguay. These are natural non-caloric sweeteners with functional and sensory properties superior to those of many synthetic high-potency sweeteners being used today [1]. For example, processed forms of stevia from a mixture of seven to nine different steviol glycosides (comprising any combinations of rebaudioside A, B, C, D, E, F, stevioside, steviolbioside, rubusoside and dulcoside A) to highly pure rebaudioside (“reb”) A can be 100 to 300 times more potent than sugar [2]. Reb D has been reported to be cleaner in its sweet taste than reb A and therefore its use as a natural high potency sweetener of future is not ruled out. Reb C on the other hand has been reported to be a sweetness enhancer [3,4]. The use of substantially pure steviol glycosides, however, is often limited or made difficult by their low aqueous solubility or lack of aqueous solubility at higher concentrations. Both reb C and D are very low in solubility <0.1–0.2% [2] and this is limiting for their use in our products. Reb A although has intrinsic solubility of 0.8% but if it is in its hydrate form, it does not solubilize in water at all (~0.1%, based on our in-house experiments).

**Figure 1:**
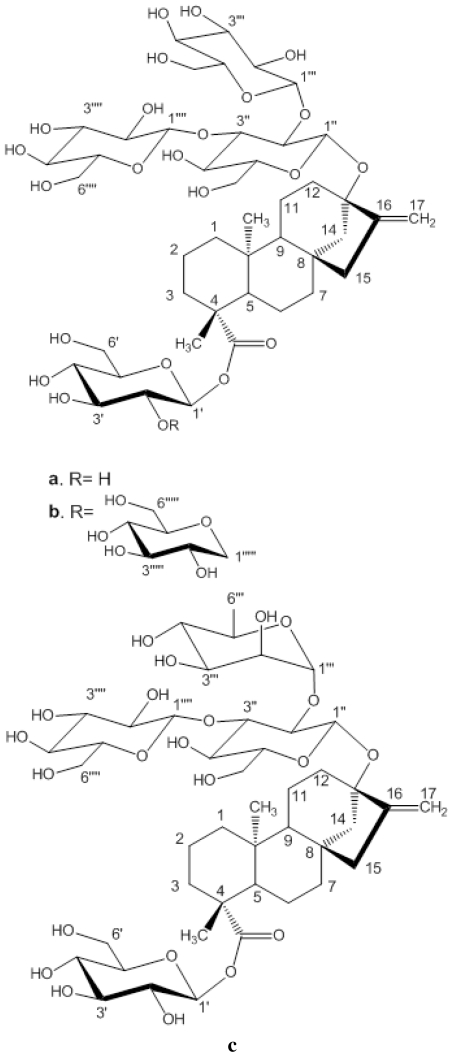
Structures and carbon numberings of the steviol glycosides (**a**) rebaudioside A (**b**) rebaudioside D (**c**) rebaudioside C.

There are several approaches used in industry towards enhancing aqueous solubility of an ingredient such as use of co-solutes, salts, solvent etc. To improve the solubility and dissolution properties of poorly soluble compounds or polymorphs, inclusion complex formation with cyclodextrin (CD) is a very widely used strategy [5,6]. These are cyclic oligosaccharide molecules of natural origin resulting from starch degradation by cycloglycosyl transferase amylases (CGTases) produced by various bacilli e.g., Bacillus circulans [7]. They have six, seven, or eight alpha (1,4)-linked D(+) glucopyranose units and are respectively called α-, β- and γ-CD. They generally form a toroid shape with an interior cavity that is less hydrophilic than the cyclodextrin exterior because of the presence of hydroxyl groups. Steviol glycosides have active hydrophilic (glycoside) and hydrophobic (diterpene) groups (Figure 1). In an aqueous solution water molecules inside the CD cavity can be replaced by apolar parts of molecules leading to an inclusion host-guest complex. In such inclusion complexes cyclodextrin host thus modifies physico-chemical properties of the included guest molecule such as its physical state, stability, solubility and bioavailability. In present study solid γ-CD/steviol glycosides, reb A, C and D were prepared by freeze drying method. We observed that upon complexation the guest steviol glycosides have higher water solubility. These cyclodextrin inclusion complexes have been characterized in solid state using techniques, including cyclic differential scanning calorimetry (DSC), Fourier transform infrared spectroscopy (FT-IR), Raman and 13C CP/MAS NMR and in solution using proton NMR and phase solubility studies. The γ-CD/reb D-(B) inclusion complex was treated with homogenizer before freeze drying to see if the stabilizing interactions get disrupted with the exercise and how different are the results compared to γ-CD/reb D-(A) inclusion complex. These studies provided a clear indication of the molecular level interactions between the CD host and guest molecules. This is the first report of the characterization of inclusion complexes of steviol glycosides. These steviol glycoside/CD inclusion complexes have strong commercial potential as novel additives in sweetener compositions of non-caloric or low caloric beverage products.

## 2. Results and Discussion

The steviol glycosides inclusion complexes were first subjected to preliminary solubility analysis which indicated that upon complexation the guest steviol glycosides have higher water solubility. The results are depicted in Table 1 below.

Further spectroscopic studies were carried out on solid inclusion complexes in comparison with the corresponding physical mixture and the individual components.

### 2.1. Differential Scanning Calorimetry (DSC) Study

**Figure 2:**
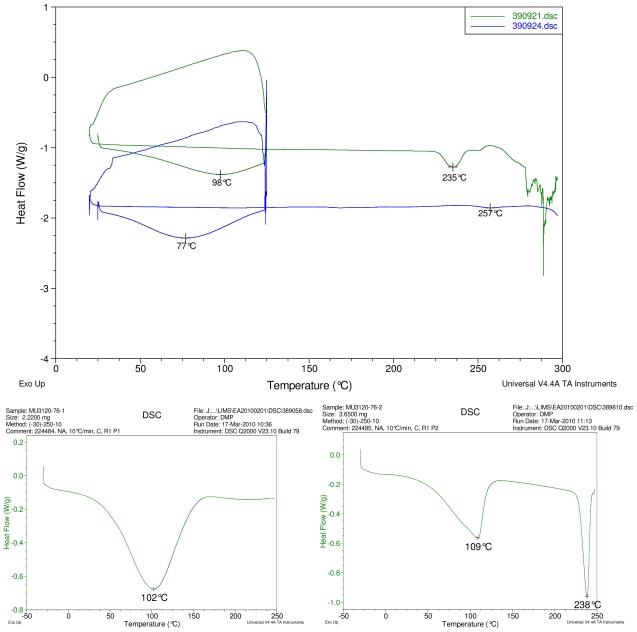
At top, overlay of DSC thermograms of physical mixture of γ-CD and reb A, top trace, green and inclusion complex γ-CD/reb A bottom trace, blue. At bottom, differential scanning calorimetry (DSC) thermograms of cyclodextrin, left and reb A, right.

**Figure 5:**
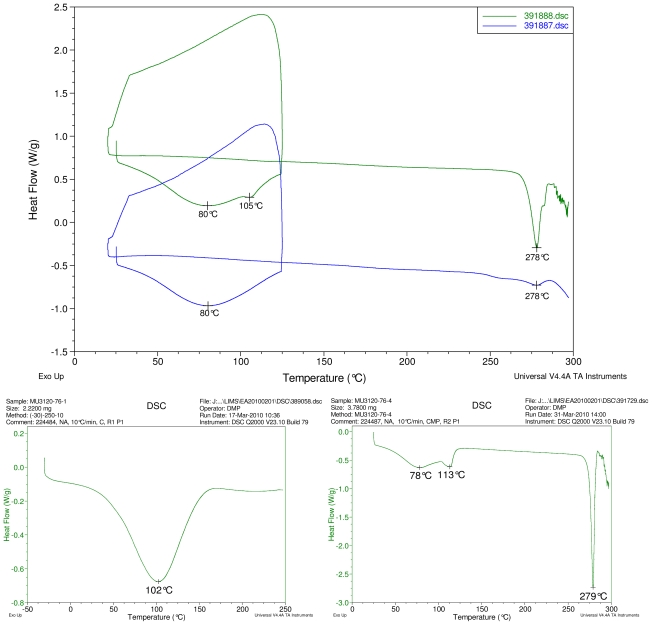
At top, overlay of DSC thermograms of physical mixture of γ-CD and reb D, top trace, green and inclusion complex γ-CD/reb D-(B) bottom trace, blue. At bottom, DSC thermograms of cyclodextrin, left and reb D, right.

Figure 2–Figure 5 display results of DSC analyses. The top plot in each figure displays an overlay of the thermograms of an inclusion complex and the corresponding physical mixture of components. The thermogram of cyclodextrin is displayed at bottom left in each figure. The thermogram of the steviol glycoside is displayed at bottom right in each figure. Temperatures of selected thermal events are labeled on the DSC traces.

The first heating cycle for each sample displays a broad endotherm spanning from ambient temperature to the end of cycle near 125 °C, consistent with loss of adsorbed water from the hygroscopic samples. An overlapping endothermic peak is observed near 100 °C in the thermograms of physical mixture samples of γ-CD with reb C, and reb D respectively. This second thermal event near 100 °C has not been assigned for the physical mixtures, though its endothermic character suggests it may potentially be attributable to the presence of crystalline material in the samples (for example, a solid-solid transition or melt), or an enthalpic relaxation of the amorphous material.

A similar peak near 100 °C is observed in the thermograms of each of the steviol glycosides, allowing for the possibility that some of the sample material may have crystallized either prior to the analyses, or during the DSC run. The absence of the endothermic peak near 100 °C in the thermograms of the inclusion complexes is consistent with the presence of a stabilizing interaction hindering crystallization of the steviol glycosides, whether occurring during or prior to the DSC analyses. Crystallization exotherms are undetected in the physical mixture sample data, although they may be obscured by the broad desolvation event or the overlapping endothermic event near 100 °C present for various samples. Crystallization exotherms are also not observed in the inclusion complex sample data. The second heating cycle for each physical mixture displays a strong endothermic peak above 200 °C (below decomposition), which is similar in temperature to a peak assigned to melting in the thermogram of each corresponding steviol glycoside. In contrast, the second heating cycles for the inclusion complexes display only broad, relatively weak, thermal events prior to decomposition. The absence of a strong endothermic melting peak above 200 °C in the thermograms of the inclusion complexes suggests the presence of a stabilizing interaction, hindering crystallization of the amorphous steviol glycosides [8].

Only the thermogram of inclusion complex γ-CD/reb D-(B), displays a non-negligible peak at the expected melting temperature indicating that some of the stabilizing interaction disappears during the homogenization process resulting in small amount of reb D crystallization as evidence of a possible phase impurity (Figure 5).

### 2.2. Fourier Transform Infrared Spectroscopy (FT-IR) Study

The infrared spectra of cyclodextrin and the steviol glycosides were corrected for presence of water vapor and intensity was normalized. Linear combinations of IR spectra were generated using cyclodextrin and each steviol glycoside; each component spectrum was arbitrarily scaled to produce an addition spectrum that closely resembles the corresponding physical mixture spectrum. These addition spectra were overlaid with the infrared spectra of the corresponding supplied physical mixtures and both were found to match well.

**Figure 6:**
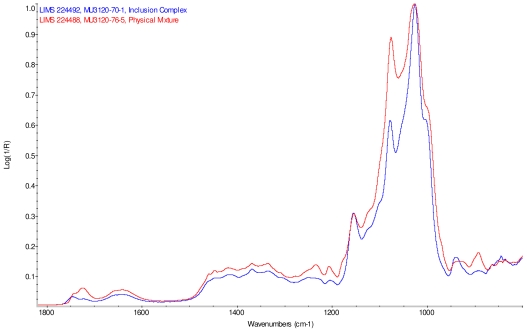
Comparison of IR spectra of physical mixture of γ-CD with reb A, red and inclusion complex γ-CD/reb A, blue.

**Figure 9:**
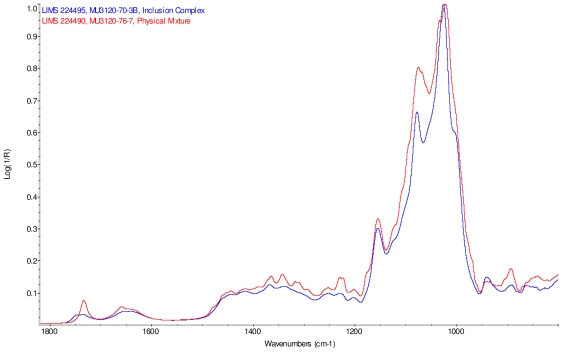
Comparison of IR spectra of physical mixture of γ-CD with reb D, red and inclusion complex γ-CD/reb D-(B), blue.

Intensity normalized infrared spectra of each inclusion complex with its corresponding physical mixture are overlaid as displayed in Figure 6–Figure 9. Infrared spectra of the inclusion complexes and corresponding physical mixtures display clear variations in band positions and intensities, indicating differences in solid state compositions of each sample set.

IR Spectra for inclusion complex samples γ-CD/reb A, γ-CD/reb C and γ-CD/reb D-(A) display the steviol glycoside carbonyl band near 1750 cm^−1^ with greater relative intensity than a weaker shoulder band near 1730 cm^−1^. In contrast, infrared spectra of physical mixture samples of γ-CD and reb C, γ-CD and reb D display only a single band near 1730 cm^−1^; the spectrum of physical mixture sample γ-CD and reb A displays both bands in the carbonyl region, but the 1750 cm^−1^ band appears as a shoulder to the more intense 1730 cm^−1^ band. Distinctive spectral features assigned to cyclodextrin vibrational modes were noted in the data. For example, in the spectra of cyclodextrin and the physical mixtures samples, the strongest C–O stretching band is present at 1026 cm^−1^; however, the band is shifted to 1023 cm^−1^ in the spectra of the inclusion complexes. Similarly, the weak band at 1150 cm^−1^ in the spectra of cyclodextrin and the physical mixtures samples is shifted to 1155 cm^−1^ in the spectra of the inclusion complexes. Other notable differences in the spectra of the inclusion complexes and corresponding physical mixtures include the markedly reduced intensity of the 1080 cm^−1^ band in the spectra of the inclusion complexes, and differences in shape and relative intensities for both the broad band in the hydroxyl stretching region and the series of bands in the CH stretching region. The observation of shifted bands and anomalous intensities in the spectra of the inclusion complexes is consistent with the anticipated presence of an interaction between cyclodextrin and the steviol glycosides.

X-ray powder diffraction (XRPD) analysis was later performed on γ-cyclodextrin sample to confirm its amorphous character. However, the observation of band shifting for vibrations assigned to cyclodextrin further supports the hypothesis of a stabilizing interaction present in the inclusion complexes [9]. Further XRPD testing was not performed on the remaining samples.

**Figure 10:**
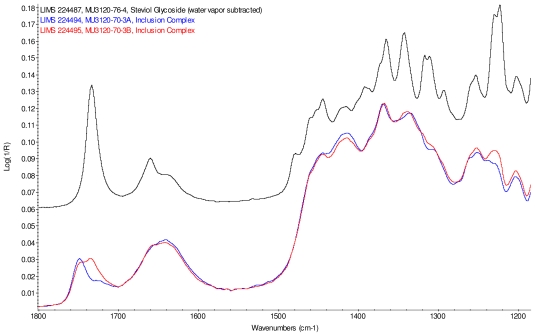
Comparison of IR spectra of inclusion complex γ-CD/reb D-(A), (lower trace, overlaid, blue), inclusion complex γ-CD/reb D-(B) (lower trace, overlaid, red), and rebaudioside D (top trace, black).

Figure 10 displays an overlay of the infrared spectra of inclusion complexes γ-CD/reb D-(A) and γ-CD/reb D-(B) passed through a homogenizer, and the spectrum of steviol glycoside reb D. Differences in band intensities are evident in various regions of the spectra. The spectral regions displaying intensity differences correspond to band frequencies in the spectrum of reb D, suggesting the presence of a phase impurity of this steviol glycoside in the sample.

### 2.3. Raman Spectral Studies

Raman spectra were treated similar to the infrared data. The calculated addition spectra were found to match well to the corresponding physical mixture spectra. Raman spectra of the physical mixture and inclusion complex samples were captured after both 256 and 512 scans during data acquisition, to investigate the effect of the Raman laser on the integrity of the samples. Only minor differences were observed between the two spectra for each sample, with the exception of physical mixture samples of γ-CD with reb C and with reb D. Evaluation of Raman data was therefore carried out using the spectra acquired after 256 accumulations for all samples.

**Figure 11:**
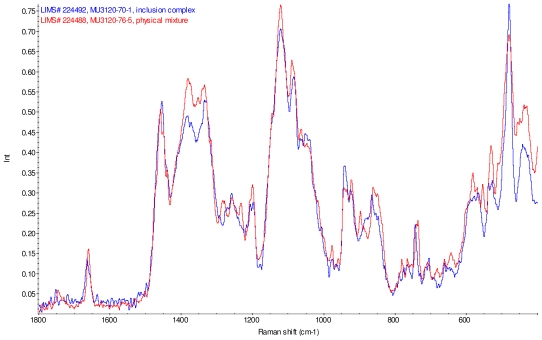
Comparison of diagnostic region of Raman spectra of physical mixture of γ-CD with reb A, red and inclusion complex γ-CD/reb A, blue.

Figure 11–Figue 14 display overlays of the intensity normalized Raman spectra of each inclusion complex with its corresponding physical mixture. Variations in band positions and intensities are observed between the Raman spectra of the inclusion complexes and corresponding physical mixtures, consistent with differences in solid state compositions of each sample set. For example, each physical mixture spectrum displays weak peaks near 1280 and 1230 cm^−1^ which are absent in the spectra of the inclusion complexes, with the exception of γ-CD/reb D-(B) which displays only very weak peaks at these frequencies. Also, the peak near 1660 cm^−1^ assigned to C=C stretching of the steviol glycosides is shifted 4 cm^−1^ to higher frequency in the spectrum of inclusion complex sample γ-CD/reb A, and is broadened in the spectra of inclusion complex samples γ-CD/reb D-(A) and γ-CD/reb D-(B). Further differences between the Raman spectra of the inclusion complexes and corresponding physical mixtures include: the relatively narrower shape of the cyclodextrin peak near 480 cm^−1^ in the spectra of the inclusion complex samples; and the appearance of a single sharp peak at 743 cm^−1^ in the spectrum of each inclusion complex sample, in contrast to the one or more peaks present in this region with variable width and frequency in the spectra of the physical mixtures.

### 2.4. NMR Spectroscopy Studies

#### ^1^H NMR

**Figure 15:**
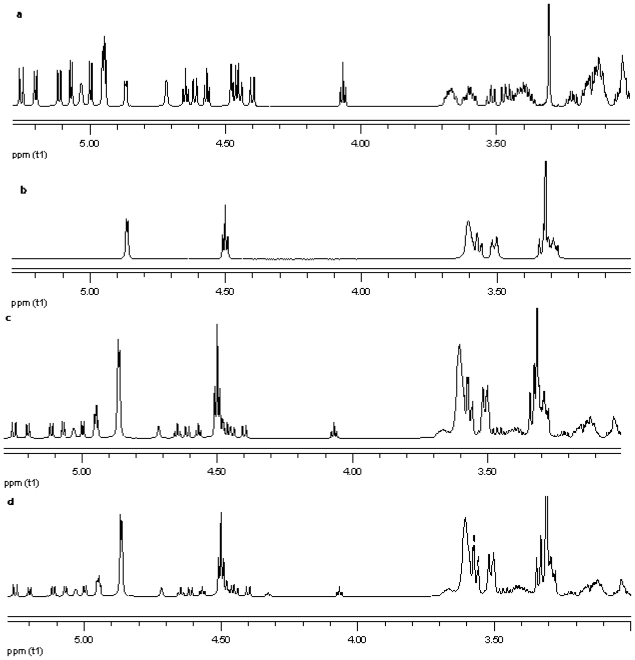
Proton NMR data from top (**a**) reb A; (**b**) γ-CD; (**c**) physical mixture of γ-CD and reb A; and (**d**) inclusion complex γ-CD/reb A.

**Figure 16:**
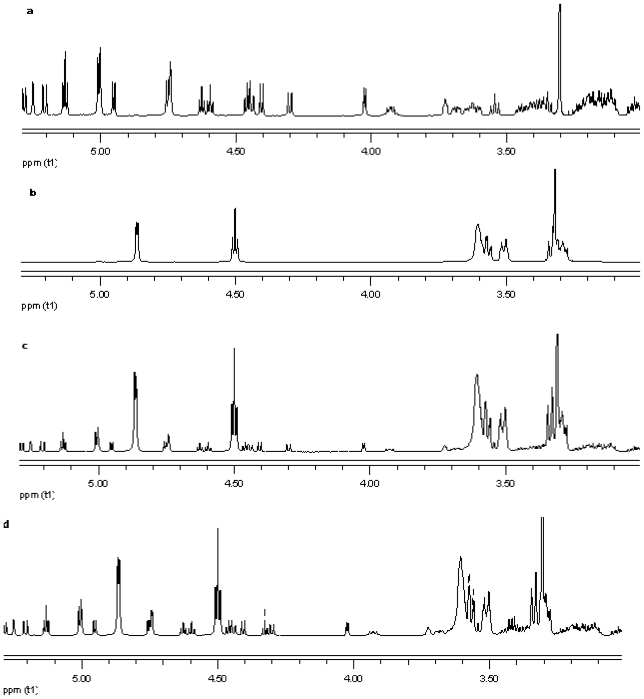
Proton NMR data from top (**a**) reb C; (**b**) γ-CD; (**c**) physical mixture of γ-CD and reb C; and (**d**) inclusion complex γ-CD/reb C.

**Figure 17:**
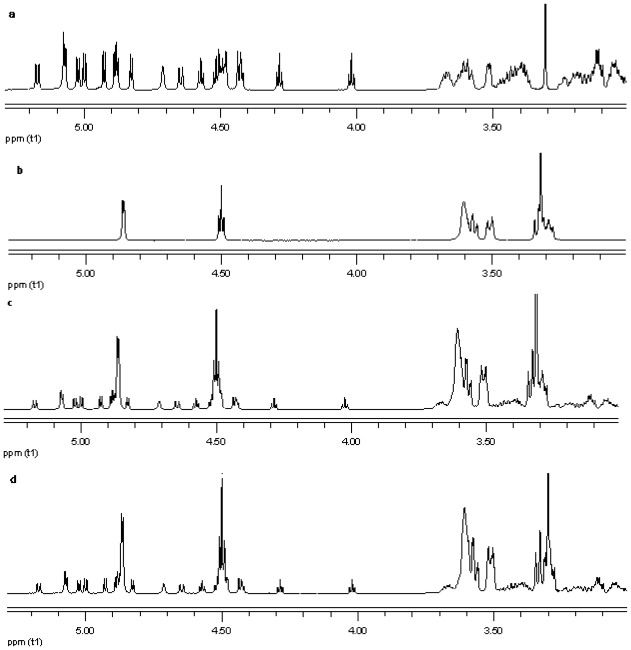
Proton NMR data from top (**a**) reb D; (**b**) γ-CD; (**c**) physical mixture of γ-CD and reb D; and (**d**) inclusion complex γ-CD/reb D.

NMR spectroscopy is an important tool for physicochemical characterization of inclusion complexes between cyclodextrin and guest molecules. In present study comparison of the proton NMR spectra of the equimolar physical mixture of the steviol glycoside with cyclodextrin and the corresponding inclusion complex shows only minor changes (see Figure 15–Figure 17). The steviol glycoside proton NMR typically has the steviol backbone protons from 0.5 to 2.5 PPM, then nonanomeric sugar protons from 3 to 4 PPM and then anomeric protons in the region of 4.5 to 5.5 PPM. As DMSO-d6, an aprotic solvent has been used as NMR solvent here, the NMR signals of the hydroxyl protons also appear along with anomeric protons. In case of reb A there are minor peak changes around 3.3, 4.5 and 4.95 PPM in the inclusion complex versus the physical mixture (Figure 15). There is a small new triplet at 4.33 PPM in the spectra of the inclusion complex while it is not present in the physical mixture or in any of the components alone. Similar is the case with reb C, a more prominent new triplet peak appears at 4.326 PPM in the spectra of the inclusion complex while it is not present in the equimolar mixture or in any of the components (Figure 16). In case of reb D there are minor changes in peaks around 3.3 PPM in the inclusion complex versus the physical mixture (Figure 17). The H-3 proton of gamma cyclodextrin which is located inside the cyclodextrin cavity shows a minor upfield shift in all the three inclusion complexes from 4.502 in gamma CD spectra to 4.500 in reb A/*γ*-CD and reb C/*γ*-CD while 4.499 PPM in reb D/*γ*-CD inclusion complex. The H-5 proton of gamma cyclodextrin which is also located inside the cyclodextrin cavity shows a minor downfield shift in all the three inclusion complexes from 3.566 in gamma CD spectra to 3.567 in reb A/*γ*-CD, 3.568 in reb C/*γ*-CD while 3.569 PPM in reb D/*γ*-CD inclusion complex.

#### ^13^C CP/MAS Solid state NMR

**Figure 18:**
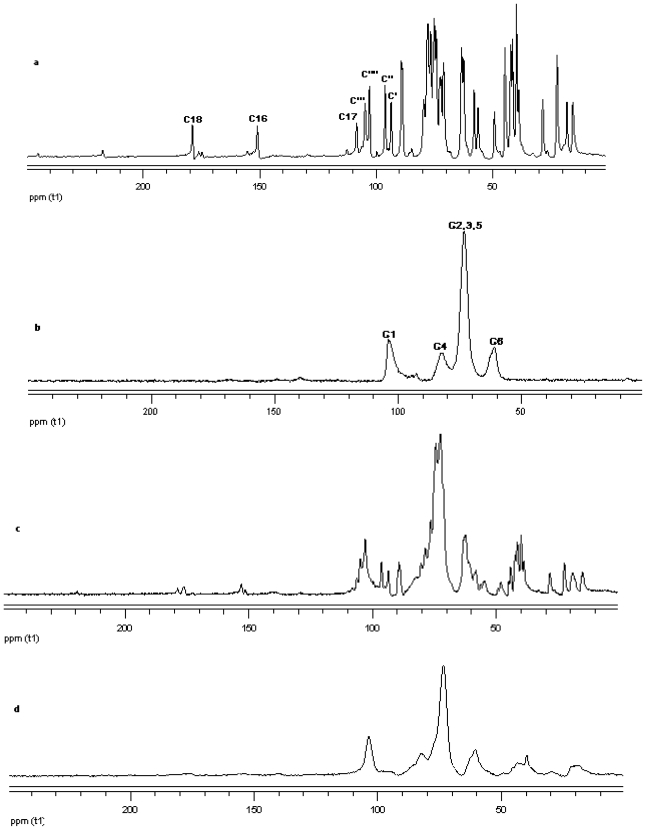
^13^C CP/MAS solid state NMR data from top (**a**) reb A; (**b**) γ-CD; (**c**) physical mixture of γ-CD and reb A; and (**d**) inclusion complex γ-CD/reb A.

**Figure 19:**
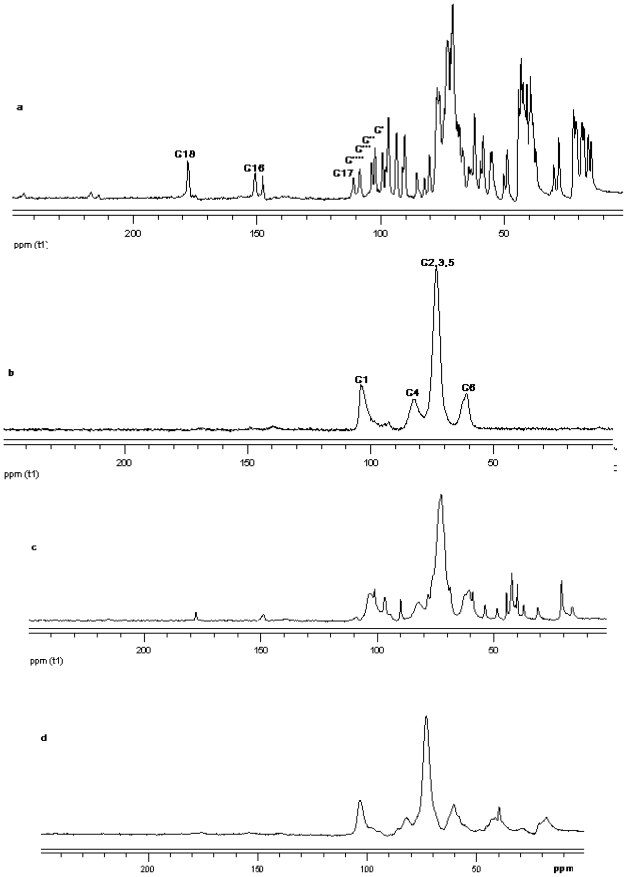
^13^C CP/MAS solid state NMR data from top (**a**) reb C; (**b**) γ-CD; (**c**) physical mixture of γ-CD and reb C; and (**d**) inclusion complex γ-CD/reb C.

**Figure 20:**
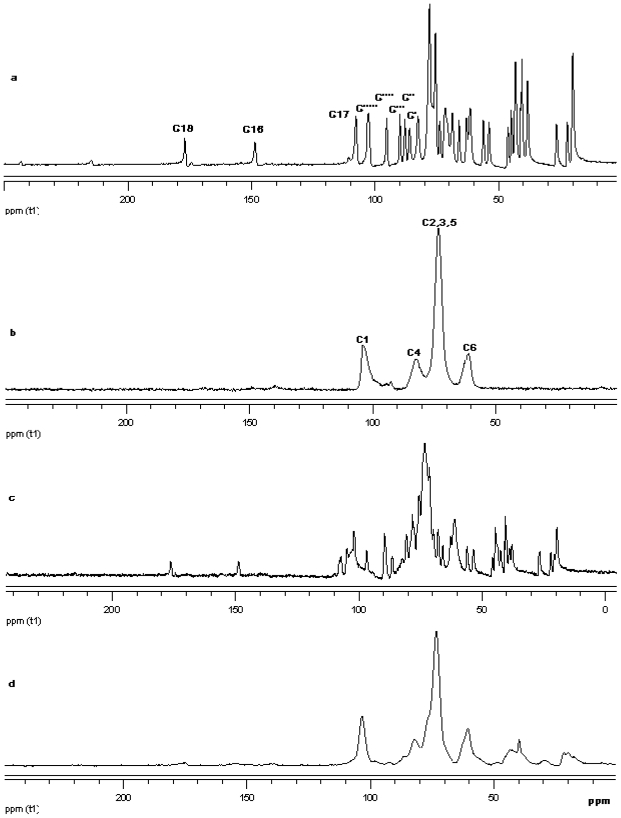
^13^C CP/MAS solid state NMR data from top (**a**) reb D; (**b**) γ-CD; (**c**) physical mixture of γ-CD and reb D; and (**d**) inclusion complex γ-CD/reb D.

The ^13^C CP/MAS NMR spectrum of free *γ*-CD and free steviol glycoside has been compared with their corresponding physical mixture and inclusion complexes in Figure 18–Figure 20. It is clear that physical mixtures of all the steviol glycosides appeared to be an equivalent of the addition of the individual components and hence indicate lacking of any molecular level interaction. The spectrum of the inclusion complex shows most of the major resonances of reb A and gamma cyclodextrin in Figure 18. Some of the peaks especially C18 and C16 appear to be completely disappeared in the inclusion complex spectra. As compared to the physical mixture, the resonances of both reb A and gamma cyclodextrin show reduced splitting and broadening. The resonances of reb A signals are considerably more broadened as compared to the pure reb A spectrum which suggests that reb A molecules are now in a less ordered and more amorphous environment. This is consistent with a true inclusion complex formation. Similar pattern has been seen upon studying ^13^C CP/MAS NMR spectrum of reb C and reb D inclusion complexes in Figure 19 and Figure 20.

### 2.5. Phase Solubility Analysis

**Figure 21:**
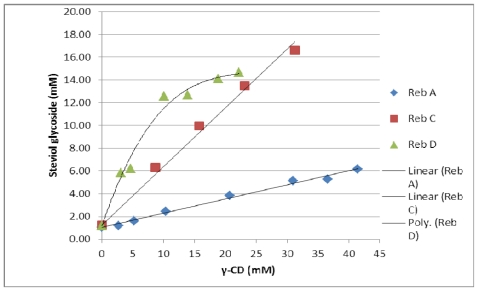
Phase solubility diagrams for the three steviol glycosides, reb A, reb C and reb D with γ-CD in aqueous solution at 25 °C.

The effect of γ-cyclodextrin on the solubility of all three steviol glycosides was investigated. The solubility of reb A is 0.8% (w/v) while that of free reb C and D are around 0.1–0.2% (w/v) [2]. The solubility profile for all three steviol glycosides showed an increase in their solubility with the increasing concentration of *γ*-CD. The phase solubility diagrams showed an apparent increase in solubility of reb A and C as a linear function of *γ*-CD concentration and the isotherm profiles are A_L_ type according to Higuchi and Connors [10]. The phase solubility diagram for reb D has a negative deviation from linearity indicating that the solubilizer is less effective at higher concentrations and this solubility profile classified as A_N_ type. It can result from an alteration in the effective nature of the solvent due to presence of large amount of *γ*-CD such as bulk property changes of the solvent like viscosity, surface tension etc. and or a self-association of *γ*-CD at higher concentrations [11]. The phase solubility diagrams for these three steviol glycosides are shown in Figure 21. The apparent K_1:1_ stability constant were calculated from the straight line of the phase solubility diagrams for reb A and reb C and from the linear section of the phase solubility diagram for reb D and the intrinsic solubilities of these steviol glycosides in water (S_0_) by using the following equations:

$$K_{1:1}=\frac{slope}{S_{0}(1-slope)}$$

Table 2 shows values of the apparent stability constant K_1:1_ and the enhancement factor (EF). The higher values of K_1:1_ for reb C and reb D as compared to reb A suggested that they formed more stable complexes with *γ*-CD then reb A. As slope is greater than one in case of both reb C and D therefore higher order complexes are expected to be involved in their solubilization.

## 3. Experimental Section

### Materials

Rebaudioside A, C and D of 97+% purity were obtained from in-house purification work on low purity stevia samples. Food grade γ-cyclodextrin was received as a sample from Wacker Fine Chemicals (Adrian, MI, USA).

### Preparation of γ-CD/Reb A (hydrate form) and γ-CD/Reb C Inclusion Complexes

Equimolar amounts of hydrate form of reb A or reb C were combined with one mmol of γ-cyclodextrin (γ-CD) and suspended in water (10 mL). The solution was heated with stirring up to 67 °C. To this was added 95% ethanol drop-wise until the solution started to become clear, within 5 minutes (1.5 to 3.0 mL). Once the solution was clear, it was cooled to room temperature and then freeze-dried for 48 hours.

### Preparation of γ-CD/Reb D Inclusion complex. A

Equimolar amounts of Reb D was combined with one mmol γ-cyclodextrin and suspended in water (30 mL), methanol (30 mL) and ethanol (10 mL) and was heated with vigorous stirring upto 60 °C for 30 minutes. Once the solution was clear, it was cooled to room temperature and then freeze-dried for 48 hours.

### B

In this case the clear aqueous alcoholic solution from above after cooling down to room temperature was passed through a homogenizer at 10 K Psi and then freeze dried. The idea was to later on see if homogenization breaks up some of the molecular interactions that stabilize the inclusion complexes.

### Preparation of Physical Mixtures

Corresponding physical mixtures of cyclodextrin with each steviol glycoside were prepared from freeze dried γ-cyclodextrin and steviol glycosides. Physical mixture of reb A and gamma cyclodextrin were prepared with equimolar amount as in solid CD inclusion complex by uniformly mixing the two components. Same was followed for reb C and reb D.

### Preliminary solubility analysis

To measure the solubility, a substantially pure terpene glycoside complexed with a cyclodextrin was combined with water with less than 1 minute of magnetic stirring. To prepare sample 1, 234.19 mg of γ-CD/Reb A (hydrate form) inclusion complex prepared was combined with water to total 7 g of solution (equivalent to 100 mg Reb A). To prepare sample 2, 473 mg of γ-CD/Reb C complex was combined with water to total 10 g of solution (equivalent to 200 mg Reb C). To prepare sample 3, the amount of inclusion complex used to prepare sample 2 was doubled. To prepare sample 4, 107.5 mg of γ-CD/Reb D complex was combined with water to total 5 g of solution (equivalent to 50.0 mg Reb D). The solutions were stirred intermittently and were monitored visually for several days.

### Differential Scanning Calorimetry

DSC was performed using a TA Instruments Q2000 differential scanning calorimeter. Temperature calibration was performed using NIST traceable indium metal. The sample was placed into an aluminum DSC pan, covered with a lid, and the weight was accurately recorded. Pan lids were manually perforated with a pinhole for all samples except for gamma cyclodextrin, rebaudioside A and rebaudioside C. A weighed aluminum pan configured as the sample pan was placed on the reference side of the cell. The data acquisition parameters and pan configuration for each thermogram are displayed in the header of the thermogram in the Data section of this report. Cyclodextrin and the steviol glycosides were heated from −30 °C to either 250 or 300 °C at 10 °C/min. Inclusion complexes and physical mixtures were heated from ambient to 125 °C at 10 °C/min, held isothermal for one minute at 125 °C, rapidly cooled to 20 °C, and then heated to 300 °C at 10 °C/min.

### Raman Spectroscopy

Raman spectra were acquired on a FT-Raman module interfaced to a Nexus 670 FT-IR spectrophotometer (Thermo Nicolet) equipped with an indium gallium arsenide (InGaAs) detector. Wavelength verification was performed using sulfur and cyclohexane. Each sample was prepared for analysis by placing the sample into a pellet holder. Approximately 0.5 W of Nd:YVO_4_ laser power (1064 nm excitation wavelength) was used to irradiate the sample. Each spectrum represents either 256 or 512 co-added scans collected at a spectral resolution of 4 cm^−1^.

### IR Spectroscopy

IR spectra were acquired on Magna-IR 860^®^ Fourier transform infrared (FT-IR) spectrophotometer (Thermo Nicolet) equipped with an Ever-Glo mid/far IR source, an extended range potassium bromide (KBr) beamsplitter, and a deuterated triglycine sulfate (DTGS) detector. Wavelength verification was performed using NIST SRM 1921b (polystyrene). An attenuated total reflectance (ATR) accessory (Thunderdome™, Thermo Spectra-Tech), with a germanium (Ge) crystal was used for data acquisition. Each spectrum represents 256 co-added scans collected at a spectral resolution of 4 cm^−1^ A background data set was acquired with a clean Ge crystal. A Log 1/*R* (*R* = reflectance) spectrum was obtained by taking a ratio of these two data sets against each other.

### Phase solubility studies

Phase solubility measurements were performed according to the method of Higuchi and Connors [10]. An excess of steviol glycoside was suspended in aqueous solutions containing from zero to 41 mM concentrations of gamma cyclodextrin. Steviol glycosides in the absence of cyclodextrin were used to determine their intrinsic solubility. The suspensions were continuously stirred while being placed in for 72 h in a 25.0 ± 0.1 °C constant temperature water bath. After the equilibrium was reached the excess steviol glycoside was removed by filtration through a 0.45 μ membrane filter (Millipore, USA). The clear solutions were diluted and were analyzed by LC-MS.

### LC-MS

Steviol glycosides were detected using Waters 2695 / ZQ LC/MS System and an ESCi source in APCI mode. The method employed single ion recording (SIR): 965 (Reb-A), 803 (Reb-B), 950 (Reb-C), and 1128 (Reb-D) in negative ion. The column used was Phenomenex Prodigy ODS(3) C18 5 μm 2.0 × 250 mm. Mobile phase was: A = 0.1% NH4OAC (13 mM) + 0.2% HOAC, B = CH3CN, 80% A–60% A over 40 min (0.25 mL/min)

### ^1^H NMR Spectroscopy

All liquid NMR spectra were acquired on a Varian INOVA600 spectrometer with a 5 mm ID probe at room temperature. About 3–4 mg Samples were dissolved in deuterium DMSO-D6. The chemical shift reference is the DMSO at 2.49 ppm. Typical parameters for ^1^H NMR spectra were 64 scans, 2.4 s relaxation delay and 32k total data point were collected, zero filled to 65536 and use 0.2 Hz for line broadening (LB) before Fourier Transform (FT).

### ^13^C CP/MAS NMR Spectroscopy

All solid state NMR spectra were acquired on a Bruker Avance 600WB solid state NMR spectrometer with a 4 mm HCN MAS Probe. Samples were packed (about 42–46 mg) into 4 mm ZrO2 rotor. Chemical shifts were calibrated with an external standard of 1-glycine at 176.40 ppm. Samples were spun at 10 kHz at room temperature. Spectra were acquired with a proton 90° pulse length of 4 μs and a ^13^C-^1^H contact time of 2 ms. The repetition delay time was 10 s, and the spectral width was 78 kHz. Free induction decays were accumulated with a time domain size of 21K data points. A square-shaped pulse was used during the cross-polarization, and a TPPM decoupling pulse sequence with a phase angle of 15° was used during the acquisition. Each sample spectrum was obtained with 5000 scans and processed with 20 Hz line broadening. ^13^C resonances were assigned in spectra based on reported data of rebaudioside A, C and D [12,13] and *γ*-CD [14].

## 4. Conclusions

Inclusion complex formation with gamma cyclodextrin of reb A, C and D have all resulted in inclusion complex formation and the physical properties of the complex have been found to be different from the starting steviol glycoside. In case of reb A experiments have been carried out with hydrate form of reb A as this form is most difficult to solubilize and converting it into soluble form can have the biggest commercial impact. Thermal and spectroscopic characterization data are consistent with the presence of a stabilizing interaction between cyclodextrin and the steviol glycosides. DSC thermograms of the inclusion complexes display an absence of melting peaks which are observed in the physical mixture samples. In addition, lower temperature thermal events, which are tentatively assigned to either enthalpic relaxation or presence of crystalline steviol glycoside material, are absent in the thermograms of the inclusion complexes. Spectroscopic data from Raman and FT-IR reveal differences in band frequencies and intensities which are consistent with differences in solid state compositions of the physical mixtures and inclusion complexes. Proton NMR spectra show only negligible changes in the ^1^H chemical shifts however ^13^C CP/MAS NMR spectrum gives clear indications of a true inclusion complex formation. Phase solubility data suggest that γ-CD is useful as a solubilizer for the development of potentially acceptable aqueous formulation of the steviol glycosides.

## Figures and Tables

**Figure 3:**
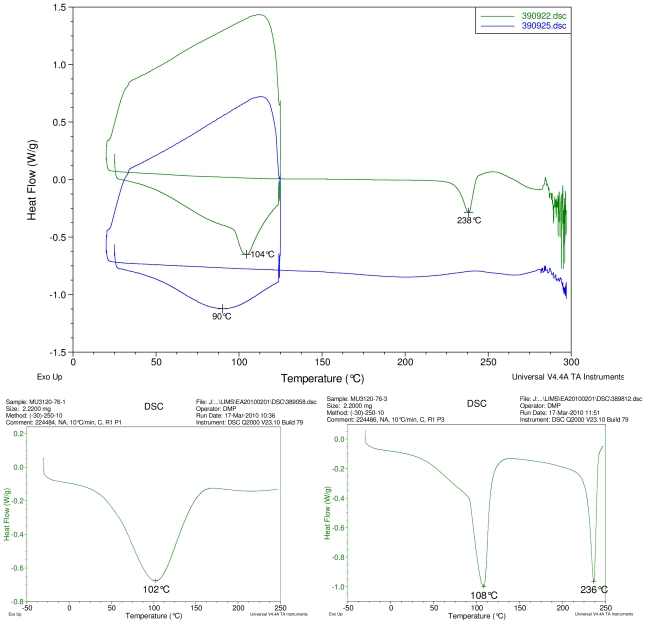
At top, overlay of DSC thermograms of physical mixture of γ-CD and reb C, top trace, green and inclusion complex γ-CD/reb C bottom trace, blue. At bottom, DSC thermograms of cyclodextrin, left and reb C, right.

**Figure 4:**
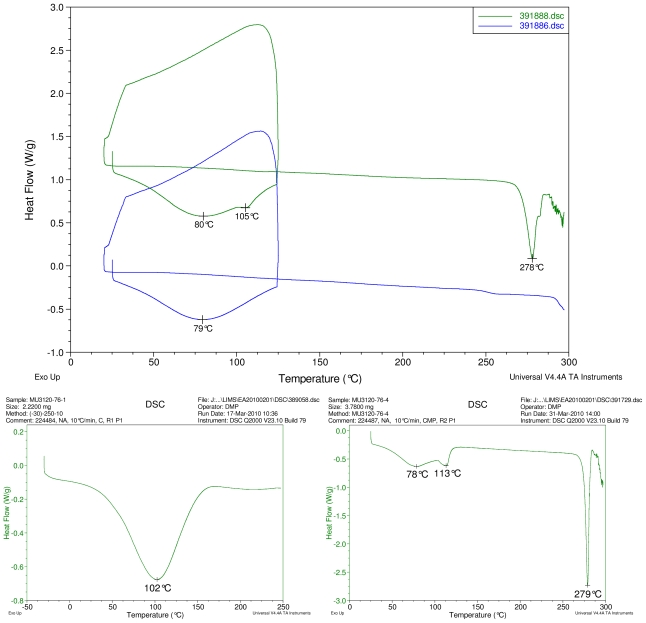
At top, overlay of DSC thermograms of physical mixture of γ-CD and reb D, top trace, green and inclusion complex γ-CD/reb D-(A) bottom trace, blue. At bottom, DSC thermograms of cyclodextrin, left and reb D, right.

**Figure 7:**
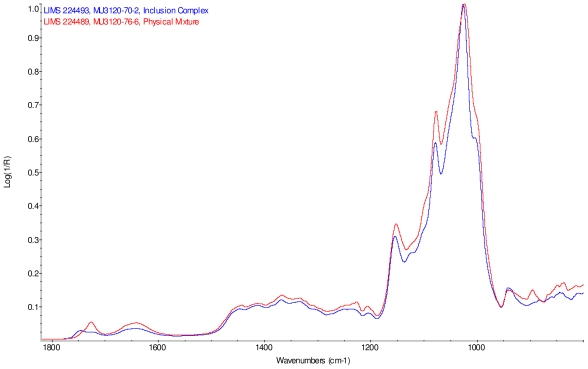
Comparison of IR spectra of physical mixture of γ-CD with reb C, red and inclusion complex γ-CD/reb C, blue.

**Figure 8:**
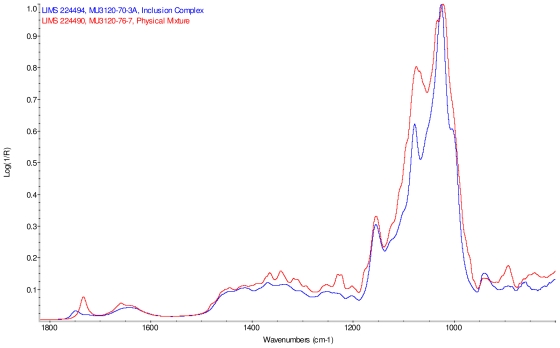
Comparison of IR spectra of physical mixture of γ-CD with reb D, red and inclusion complex γ-CD/reb D-(A), blue.

**Figure 12:**
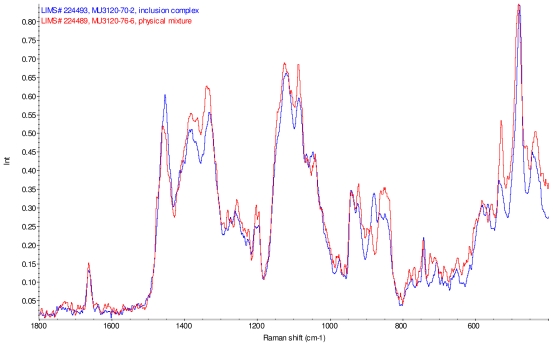
Comparison of diagnostic region of Raman spectra of physical mixture of γ-CD with reb C, red and inclusion complex inclusion complex γ-CD/reb C, blue.

**Figure 13:**
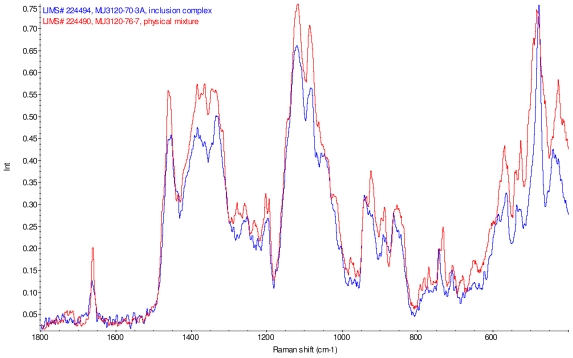
Comparison of diagnostic region of Raman spectra of physical mixture of γ-CD with reb D, red and inclusion complex γ-CD/reb D-(A), blue.

**Figure 14:**
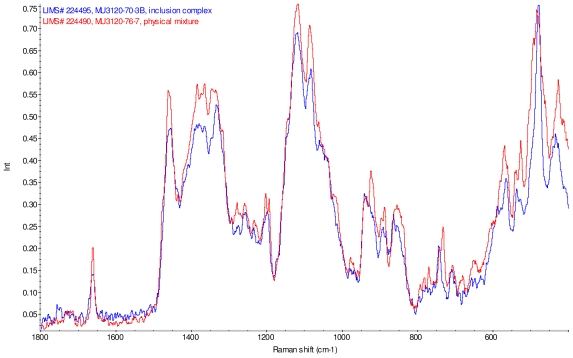
Comparison of diagnostic region of Raman spectra of physical mixture of γ-CD with reb D, red and inclusion complex γ-CD/reb D-(B), blue.

**Table 1:** Inclusion complexes preliminary solubility at 23 °C.

Sample	Inclusion Complex*	Concentration in Water	Time	Visual Observation
1	γ-CD/Reb A	1.43%	30 days	clear
2	γ-CD/Reb C	2%	30 days	clear
3	γ-CD/Reb C	4%	4 days	clear
4	γ-CD/Reb D-(A) **	1%	30 days	clear

* Free reb A is soluble in water up to 0.8% while reb C&D are soluble 0.1–0.2% [2].

** Preliminary solubility analysis of γ-CD/Reb D-(B) inclusion complex did not show any improvement over free reb D.

**Table 2:** Complex formation parameters for steviol glycoside/γ-CD systems in water at 25 °C.

Steviol glycoside	Type of diagram	Slope	K_1:1_ (M^−1^)	EF*
Reb A	A_L_	0.1242	133	12.75
Reb C	A_L_	4.9190	1028	5.5
reb D	A_N_	4.3485	1091	9.6

* The enhancement factor (EF) = S_eq_/S_0_, where S_eq_ and S_0_ are the solubilities of steviol glycoside in the presence and absence of γ-CD respectively.

## References

[1] Kim N.C., Kinghorn A.D. (2002). Sweet tasting and sweetness-modifying constituents of plants. Stud. Nat. Prod. Chem.

[2] Carakostas M., Prakash I., Kinghorn A.D., Wu C.D., Soejarto D.D., Nabors L.B. (2011). Steviol glycosides. Alternative Sweeteners.

[3] Salemme R.F., Long D., Palmer R.K., Brennan F.X., Sprous D (2011). Rebaudioside C and its stereoisomers as natural product sweetness enhancers. U.S. Patent Application.

[4] Prakash I., Dubois G.E., Klucik J., San Miguel R.I., Fritsch R.J., Chaturvedula V.S.P. (2011). Sweetness enhancers, compositions thereof and methods for use. U.S. Patent.

[5] Ohtani K., Aikawa Y., Fujisawa Y., Kasai R., Tanaka O., Yamasaki K. (1991). Solubilization of steviolbioside and steviolmonoside with gamma-cyclodextrin and its application to selective syntheses of better sweet glycosides from stevioside and rubusoside. Chem. Pharm. Bull.

[6] Szejtli J., Szente L. (2005). Elimination of bitter, disgusting tastes of drugs and foods by cyclodextrins. Eur. J. Pharm. Biopharm.

[7] Sicard P.J., Saniez M.H., Duchene D. (1987). Biosynthesis of cycloglycosyl transferase and obtention of its enzymatic reaction products. Cyclodextrins and Their Industrial Uses.

[8] Redenti E., Peveri T., Zanol M., Ventura P., Gnappi G., Montenero A. (1996). A study on the differentiation between amorphous piroxicam: Î²-cyclodextrin complex and a mixture of the two amorphous components. Int. J. Pharm.

[9] Taylor L.S., Zografi G. (1997). Spectroscopic characterization of interactions between PVP and indomethacin in amorphous molecular dispersions. Pharm. Res.

[10] Higuchi T., Connors K.A. (1965). Phase solubility techniques. Adv. Anal. Chem. Instrum.

[11] Brewster M.E., Loftsson T. (2007). Cyclodextrins as pharmaceutical solubilizers. Adv. Drug Deliv. Rev.

[12] Sakamoto I., Yamasaki K., Osamu T. (1977). Application of ^13^C NMR spectroscopy to chemistry of plant glycosides: Rebaudioside-C, a new sweet diterpene glycoside of stevia rebaudiana. Chem. Pharm. Bull.

[13] Sakamoto I., Yamasaki K., Osamu T. (1977). Application of ^13^C NMR spectroscopy to chemistry of plant glycosides: Rebaudioside D and E, new sweet diterpene glucosides of stevia rebaudiana Bertoni. Chem. Pharm. Bull.

[14] Onda M., Yamamoto Y., Inoue Y., Chujo R. (1988). ^1^H NMR study of intramolecular hdrogen bonding interaction in cyclodextrins and their di-O-methylated derivatives. Bull. Chem. Soc. Jpn.

